# Sodium Hydroxide-Free Soy Protein Isolate-Based Films Crosslinked by Pentaerythritol Glycidyl Ether

**DOI:** 10.3390/polym10121300

**Published:** 2018-11-23

**Authors:** Yingji Wu, Liping Cai, Chen Wang, Changtong Mei, Sheldon Q. Shi

**Affiliations:** 1 College of Materials Science and Engineering, Nanjing Forestry University, Nanjing 210037, China; wuyingji@njfu.edu.cn (Y.W.); liping.cai@unt.edu (L.C.); 2 Department of Mechanical and Energy Engineering, University of North Texas, Denton, TX 76203, USA; 3 Research Institute of Wood Industry, Chinese Academy of Forestry, Beijing 100091, China; wangchen0903@163.com

**Keywords:** soy protein isolate (SPI), film, sodium dodecylbenzenesulfonate (SDBS), pentaerythritol glycidyl ether (PEGE), crosslinking

## Abstract

The soy protein isolate (SPI), sodium dodecylbenzenesulfonate (SDBS) and pentaerythritol glycidyl ether (PEGE) were used to make biodegradable films in this study. Unlike the usual method that adding sodium hydroxide (NaOH) during the SPI-based film casting, SDBS was used as a surfactant playing the similar role as NaOH. Since NaOH is a chemical with corrosiveness and toxicity, the replacing of NaOH by SDBS might reduce the hazard threat during the utilization of SPI-based films in food packing application. Furthermore, the presentation of SDBS helped dispersing the hydrophobic PEGE into the hydrophilic SPI. PEGE is a crosslinking agent with multiple reactive epoxy groups. The chemical structures and micro morphologies of the fabricated films were investigated by means of FTIR, XRD, and SEM. The thermal stabilities of the films were examined by means of the thermo-gravimetric analysis. After the chemical crosslinking, the ultimate tensile strength of the film was significantly increased, meanwhile, the water absorption was dramatically decreased. It was concluded that the SPI-based film containing 4% PEGE achieved the optimal performance.

## 1. Introduction

Owing to the environmental concerns caused by the utilization of petroleum-derived synthetic polymers [1,2] and the disposal difficult materials [3,4], the products derived from renewable and sustainable resources are desired. For these reason, protein-based films for packaging and other applications are being considered to be used as a promising alternative of synthetic films [5,6]. As a side product of the soy-oil production, the soy protein isolate (SPI) has become an eco-friendly alternative to fossil products due to its inherent advantages of cost-effective, biodegradable, abundant, etc. [7,8,9,10]. However, the SPI derivatives are especially low water-resistance and poor mechanical properties that restrict their industrial applications [11,12].

Currently, many attempts have been conducted to improve the performances of films made from SPI [13], for instances, physically mixing with natural feedstocks, such as, guar gum [14], acacia gum [15], alginate [16], corn starch [17], cellulose nanocrystal [18], etc. It could also be modified by synthetic fillers, e.g., graphene [19], nanoclay [20], and carbon nanoparticles [21]; be treated by enzymes [22] and crosslinked by chemicals, e.g., dialdehyde carboxymethyl cellulose [23], methyl methacrylate [24], epoxy castor oil [25], catechol [26], and resorcinol [27]; and improve the processing methods, e.g., compression [28], extrusion [29], high-pressure homogenization [30], etc. It was suggested that the chemical crosslinking is an effective approach to significantly build up the performance of SPI-based films. From the literature review, the most common used crosslinking agents were aldehydes (e.g., glutaraldehyde, glyoxal, and formaldehyde) [31,32], phenolic derivatives [27], and epoxy compounds [25,33,34,35].

In this research, the soy protein chains were chemically crosslinked by an un-reported crosslinking agent, pentaerythritol glycidyl ether (PEGE), to improve the film performances. The multiple epoxy groups of the PEGE molecules can crosslink with SPI molecules to form a network among protein molecules so that enhancing the mechanical and water-resistance properties of the films. However, as a hydrophobic molecule, PEGE is difficult to be evenly dispersed into SPI matrix. To address this issue, a surfactant, sodium dodecylbenzenesulfonate (SDBS), was added to improve the compatibility between SPI and PEGE. Additionally, it was found that SDBS could play a similar role as the sodium hydroxide (NaOH) that is a common agent in the SPI-based film casting. The replacement of NaOH with surfactant could remain the resultant film neutral, which could greatly benefit to film users because NaOH has the major drawbacks of high corrosiveness and health risks [36].

This study was aimed at producing NaOH-free SPI-based films with neutral pH using the centrifugal casting method. A surfactant, SDBS, was employed to replace NaOH in the SPI-based film casting. The prepared SPI-based films were chemically crosslinked by PEGE. The chemical structures, thermal stabilities, and micro morphologies of the films were examined. The significate improvements of the mechanical and water-resistance properties of films by the addition of PEGE were achieved.

## 2. Materials and Methods 

### 2.1. Materials

The soy protein isolate (SPI) (>90% protein) was obtained from Sausage Maker, Inc. (Buffalo, NY, USA). Sodium dodecylbenzenesulfonate (SDBS) (technical grade) was supplied by Aldrich Chemistry (St. Louis, MO, USA). Pentaerythritol glycidyl ether (PEGE) was purchased from Chuzhou Huisheng Elec. Mater. Co. (Chuzhou, China). Glycerol (biotechnology grade) was from bioWORLD Co. (Dublin, OH, USA).

### 2.2. Film Casting

The centrifugal casting method was used for fabricating SPI-based films. The AMT-SC5052 spin caster (Affine Materials Tech., Dallas, TX, USA) with a cylindrical surface (5H × 12.7D cm^2^) was employed as the casting equipment. The feedstock solutions were composed with SPI, DI-water, glycerol, SDBS, and PEGE. The detail recipes for different films are presented in Table 1. Firstly, the feedstock solution was magnetically stirred (200 rpm) at room temperature for 1 h to obtain a homogeneous mixture. Then, the slurry-like feedstock was transferred into the spin caster and casted at 3450 rpm. After being casted for 4 h, the film was formed on the cylindrical surface of the caster. The film was torn off and placed into an oven with 105 °C for 4 h. This post treatment could help the complete reactions between SPI and PEGE. The treated films were directly used for the thermal gravimetric analysis (TGA) and water absorption tests. For other tests, the films were transferred into a conditioning container (50 ± 2% relative humidity and 20 ± 3 °C temperature) for approximate 10 d to achieve equilibrium moisture contents.

### 2.3. FTIR Analysis

The chemical structures of the fabricated films were investigated by a Thermo Scientific Nicolet 6700 Fourier transform infrared spectroscopy (FTIR, Thermo Scientific Inc., Waltham, MA, USA). An attenuated total reflectance (ATR) accessory was employed for the FTIR tests. The wavelengths ranging from 650 to 4000 cm^−1^ were applied to the FTIR analysis with an interval of 1 cm^−1^.

### 2.4. XRD Analysis

The X-ray diffraction (XRD) analysis were performed by a Shimadzu XRD-6000 meter (Shimadzu Co., Kyoto, Japan). The Cu Kα radiation (λ = 1.54060 Å) was used. The operating conditions were set to be 40 kV and 40 mA. The analysis was carried out on the X-ray continuous scanning mode. The 2*θ* range was set to be 5–60°, and the scanning speed was 2 ° min^−1^, where *θ* was the incident angle of the X-ray on the surface of samples. The XRD instrument also immediately calculated the relative crystallinity index (*RCI*) of the sample in terms of the following equation:

*RCI* = *A*_c_/(*A*_c_ + *A*_a_) × 100% (1)

where *A*_c_ = crystalline region area and *A*_a_ = amorphous region area. Three replicates were measured for each fabricated film.

### 2.5. Tensile Tests

A Shimadzu AGS-X universal testing machine (Shimadzu Co., Kyoto, Japan) was employed for the tensile tests of the fabricated films. The ASTM D882 standard was followed for the tests. For each film, seven replicates were performed. The films were cut into 18W × 120L mm^2^, which had a narrow section of 76.2L × 12.7W mm^2^. The gauge length of each specimen was 76.2 mm (3 inches). The crosshead speed of each test was set to be 50 mm min^−1^. The tensile modulus (TE), tensile strength (TS), and elongation at break (EB) of the films were determined in the tests. 

### 2.6. SEM Observation

After the gold-sputter-coating for 1 min, the tensile fracture failure cross-sections of the films were observed using an environmental scanning electron microscope (SEM) (FEI Quanta 200, FEI company, Hillsboro, OR, USA). An accelerating voltage of 15 kV was applied for the SEM observation. The SEM images were taken with a magnification of 2000×. 

### 2.7. TGA Examination

After specimens were dried to bone-dry in an oven at 105 °C and ground into powder in a mortar, the thermal gravimetric analysis (TGA) was carried out by the means of a Q50 thermogravimetric device (TA Instruments Inc., New Castle, DE, USA) with a flowing N_2_ atmosphere. The specimens were then heated to 600 °C with a ramping temperature of 10 °C min^−1^. The initial decomposition temperatures and peak temperatures at maximum degradation rate of each specimens were calculated.

### 2.8. Water Submersion Tests

From each film sized 20L × 20W mm^2^, seven replicates were used to investigate the moisture content and water absorption. To determine the water resistance of SPI-based films, 24-h water submersion tests of the films were performed according to the standard method (ASTM D1037). In addition, the moisture contents of the films were measured (ASTM D4442) after the equilibrium in the conditioning container (50 ± 2% relative humidity and 20 ± 3 °C temperature) for 10 days.

### 2.9. Statistical Analysis

The ANOVA test was used for analysing the significance between two values. It was carried out in Microsoft Excel. A Duncan’s multiple-range test (*P* = 0.05 level) was applied to compare the differences of mechanical properties and water absorption among the specimens [37]. It was conducted by XLSTAT (Addinsoft XLSTAT company, New York City, NY, USA) statistical software.

## 3. Results and Discussion

### 3.1. Chemically Crosslinking of Films

**Figure 1:**
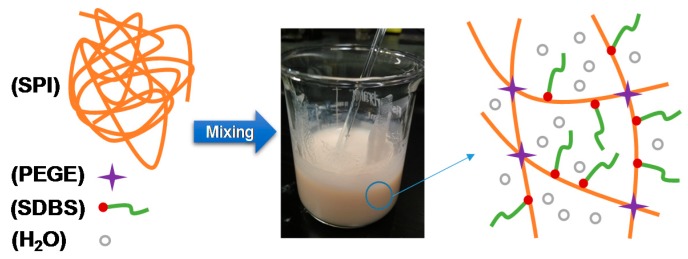
Cross-linking reaction mechanism of SPI and PEGE with the presentence of SDBS.

**Figure 2:**
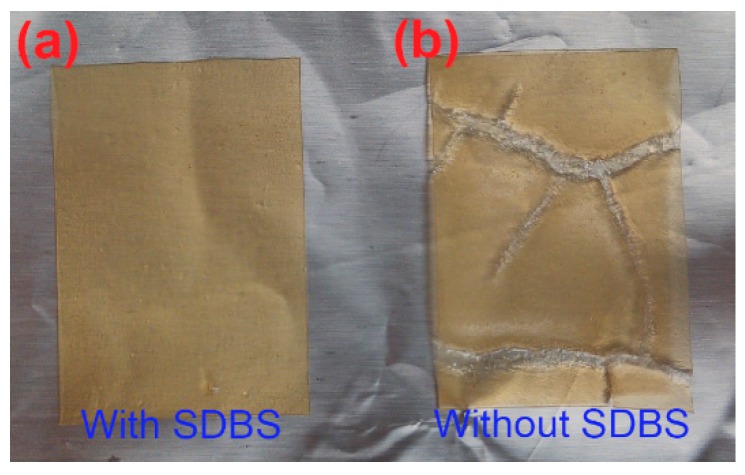
Comparison of the SPI/SDBS/PEGE4% (**a**) and SPI/PEGE4% films (**b**).

Soy protein contains abundant of amino groups [38] that can be reacted with the epoxy groups [35] on the PEGE molecules by means of the ring-opening polymerization. The crosslinking reactions between SPI and PEGE are illustrated in Figure 1. Protein and PEGE own abundant amino and four epoxy groups, respectively, which are easily reacted with each other to crosslink these two feedstocks, resulting in a chemically crosslinked film. To evaluate the effect of the SDBS addition, the images of the SPI/SDBS/PEGE4% and SPI/PEGE4% films are compared in Figure 2. The SPI/SDBS/PEGE4% film showed homogeneous, while the defects on SPI/PEGE4% film without SDBS were obvious, indicating the poor PEGE spreading in protein matrix. The similar phenomenon was observed in the preparation of the SPI/epoxidized soybean oil (ESO) [35]. In other words, the compatibility between hydrophilic protein and hydrophobic PEGE was significantly improved by SDBS because that is a recognized surfactant [39].

**Figure 3:**
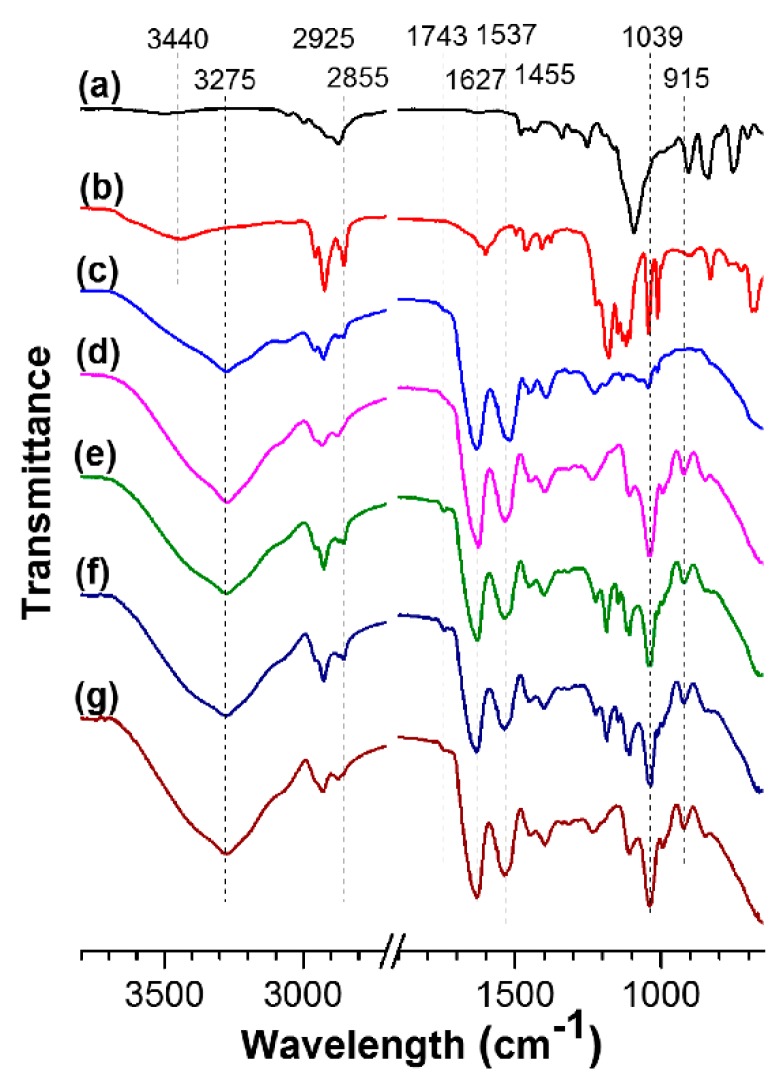
FTIR spectra of PEGE (**a**), SDBS (**b**), SPI powder (**c**), and films of SPI (**d**), SPI/SDBS (**e**), SPI/SDBS/PEGE4% (**f**), and SPI/PEGE4% (**g**).

The FTIR spectra of the feedstocks (i.e., SPI powder, SDBS, and PEGE) and films are displayed in Figure 3, in which, the peak at ~3440 cm^−1^ is contributed to the absorbed free water of SDBS. The broad peak observed at ~3275 cm^−1^ is belonged to the O–H/N–H (both free and bounded). After SPI was incorporated into films, it was observed an increase in intensity at 3275 cm^−1^, due to the introduction of plentiful –OH from glycerol. It was also observed that the characteristic C–H stretched at 2855/2925 cm^−1^ and bended at 1455 cm^−1^ from the saturated CH_2_/CH_3_. Regarding the FTIR spectrum of the SPI/SDBS, the explanation basically could be the same as that was given for SPI films. However, a clear increase in intensity of the absorption band associated with C–H stretching (2855/2925 cm^−1^) was observed due to the dodecyl chain of SDS. The relevant peaks in the FTIR spectra of SPI film exhibited at 1627, 1537 and 1233 cm^−1^, which were characteristics of O−H and N–H, C=O stretching vibration of amide I, N–H deformation of amide II, and C–H and N–H stretching of amide III, respectively. The reported FTIR peaks of amide I, II, and III from Ciannamea, et al. supported this result [40]. The C–O stretching caused the peak at 1039 cm^−1^. The FTIR absorptions of the SPI film at 3275 and 1039 cm^−1^ raised in comparison with the SPI powder, because of the existing glycerol in the film. The FTIR absorption at 1742 cm^−1^ was corresponding to the C=O stretching. The peak intensity at 1742 cm^−1^ turned into stronger after adding SDBS (Figure 3d,e), owing to the amphiphilicity of SDBS, which could help dispersing C=O to the surface of the film. The similar finding was observed after adding sodium dodecyl sulfate (SDS) [41], evidencing that the protein structural changes were promoted by SDS. The obvious peak at 915 cm^−1^ was found in SPI film, corresponding to the deformation vibration of –CH=CH_2_, which was also observed in the FTIR spectrum of PEGE (Figure 3a) owning to its epoxy groups [35]. The overlap of these two peaks resulted in the difficulty for determining the reaction degree of epoxy groups in the film, which presented the strengths near 915 cm^−1^ (Figure 3e–g). 

### 3.2. Thermal Stability of Films

**Figure 4:**
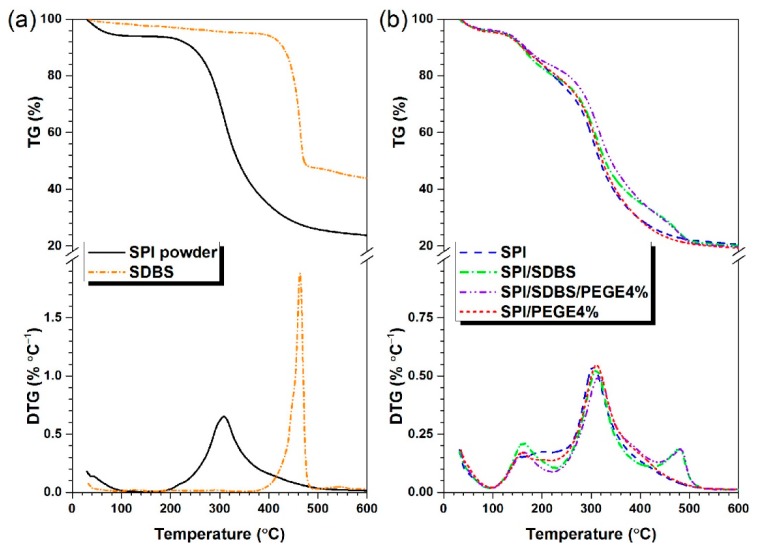
TG and DTG curves of the feedstocks, i.e., SPI powder and SDBS (**a**); and fabricated films, i.e., SPI, SPI/SDBS, SPI/SDBS/PEGE4%, and SPI/PEGE4% (**b**).

It was discovered that, after the crosslinking, the thermal and chemical stability of films increased [42]. The thermal gravimetric (TG) and the differential TG (DTG) curves of the feedstocks (SPI powder and SDBS) and fabricated films (SPI, SPI/SDBS, SPI/SDBS/PEGE4%, and SPI/PEGE4%) were examined at the temperature ranging from ~25 to 600°C, as shown in Figure 4 and Table 2. It was found that the glycerol evaporation and protein thermal decomposition occurred between 100 and 220 °C, and between 250 and 450 °C, respectively. The weight loss between 450 and 500 °C contributed to the SDBS thermal degradation (Figure 4a). The glycerol evaporation temperatures (T_max1_ in Table 2) of the SPI and SPI/PEGE4% films were 152.2 and 156.4 °C, respectively, which was slightly increased (4.2 °C) by the adding of PEGE (Figure 4b). However, the T_max1_ of SPI/SDBS and SPI/SDBS/PEGE4% films were 163.3 and 162.2 °C, respectively, which presented an increase of approximate 10 °C (Table 2). It was most likely attributed to the existing of SDBS, which helped the compatibility of protein and glycerol that deferred their evaporation [41]. This behavior confirmed the results of the enhancement of FTIR peak intensity at 1742 cm^−1^ after adding SDBS, which helped the dispersion. The protein degradation peak temperatures (T_max2_ in Table 2) of the SPI, SPI/SDBS, SPI/SDBS/PEGE4%, and SPI/PEGE4% films were 303.2, 310.9, 316.9, and 311.0 °C, respectively. T_max2_ increased 7.7 °C by adding SDBS into the SPI film, and furtherly increased 6.0 °C by introducing 4% of PEGE (Table 2). If only the crossing agent PEGE was added, the T_max2_ of film showed less increment than that modified by the combination of SDBS and PEGE. From the increase of T_max1_ and FTIR peak intensity at 1742 cm^−1^ after adding SDBS, it was learned that SDBS could help the dispersion of protein, therefore, the interactions among the protein chains were enhanced, leading to higher T_max2_ of the films. Moreover, the increase of T_max2_ after adding PEGE effectively illustrated the successful crosslinking reaction between SPI and PEGE. In conclusion, the thermal stability of the film was dramatically improved by introducing SDBS and PEGE, because of their effects of physically dispersing and chemically crosslinking the protein chains, respectively. This finding was fully consistent with the FTIR evidence.

### 3.3. Crystallinity of Films

**Figure 5:**
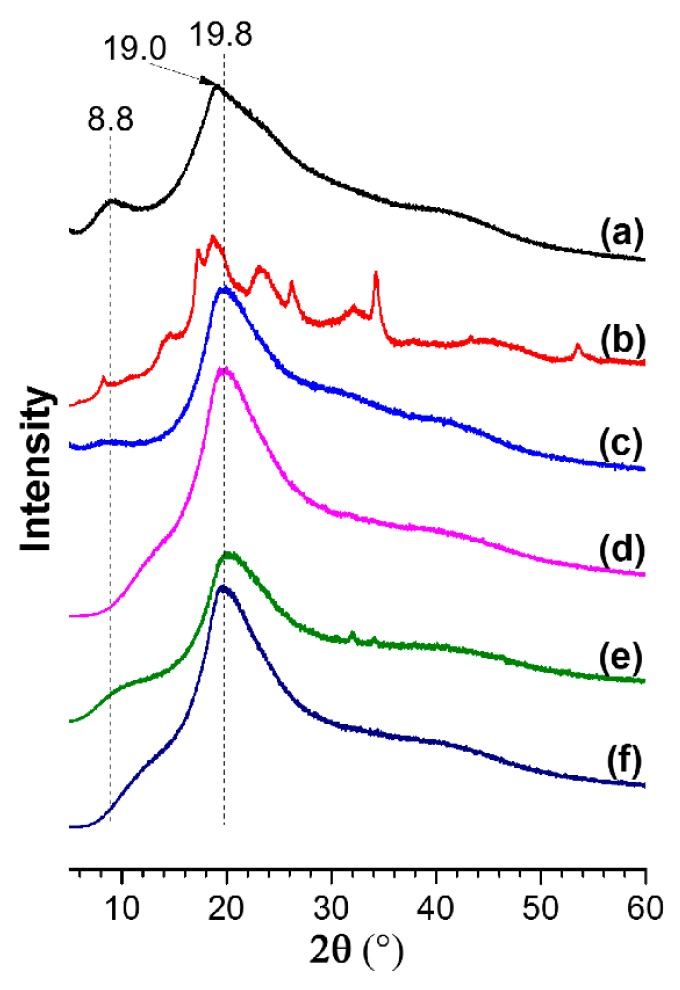
XRD patterns of SPI powder (**a**), SDBS (**b**), and films of SPI (**c**), SPI/SDBS (**d**), SPI/SDBS/PEGE4% (**e**), and SPI/PEGE4% (**f**).

Figure 5 shows the XRD patterns of the feedstocks (i.e., SPI powder, and SDBS) and films, in which the peaks at around 8.8° and 19.8° represented the α-helix and β-sheet of protein secondary structures, respectively [10,43]. In comparison with the SPI powder (19.0°), the β-sheet XRD peaks of the films were slightly increased, since the lattice constant was reduced by the addition of glycerol in the films. According to Equation (1), the values of *RCI*s were obtained (Table 3). As shown in the table, the SPI/SDBS/PEGE4% film (*RCI* = 21.0%) had the lowest *RCI* value, counting a reduction of 29.0% in comparison with the SPI film (29.5%). A significant reduction was presented from the ANOVA test (*α* = 0.01, *P* = 2.4 × 10^−3^). The crystallinity of SPI/SDBS/PEGE4% film decreased significantly, because the protein chains were not able to move freely after being crosslinked [10]. The *RCI* of SPI/SDBS and SPI/PEGE4% films presented to be 31.9% and 29.4%, respectively, which were close to *RCI* of the SPI film. It was indicated that the crystallinity of soy protein was not changed after the addition of SDBS or PEGE individually. The presentation of amphiphilic surfactant is necessary for the successful crosslinking between SPI and PEGE.

### 3.4. Micromorphology Study of Films

**Figure 6:**
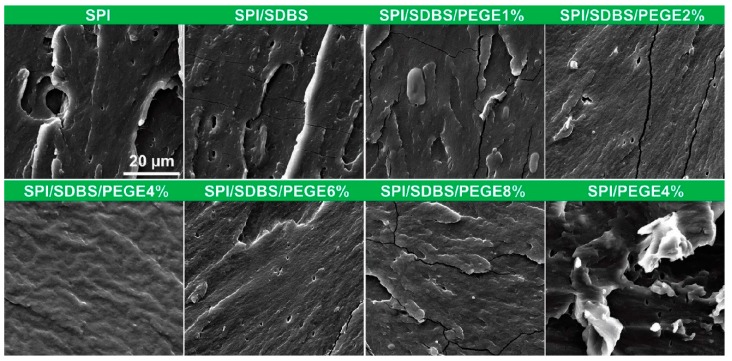
SEM images of cross-sections of the SPI-based films.

Figure 6 shows the cross-sectional morphologies of the films after tensile tests, in which, the defects and inhomogeneous characteristics were observed in the SPI film. After SDBS was introduced, the surface was not dramatically improved, remaining with obvious large pores and fracture surfaces. The SPI/SDBS/PEGE films showed relatively homogeneous surfaces and the SPI/SDBS/PEGE4% films presented the most homogeneous surfaces with fewest defects, i.e., almost non-pore and inconspicuous broken surface. As a comparison, the defects of SPI/SDBS/PEGE films with 2% and 6% PEGE were obvious, probably depending on the ratio of reactive groups on the soy proteins and PEGE. In other words, the SPI with 4% PEGE might react with each other more complete than those with other contents of PEGE. Furthermore, compared to the SPI/SDBS/PEGE4% film, the film without SDBS (the SPI/PEGE4% film) contained numerous defects. It was tallied with the macro images (Figure 2), which was caused by the heterogeneous dispersion of PEGE in the SPI matrix. The SEM results revealed that the relatively homogeneous surfaces were observed on the SPI/SDBS/PEGE films, owning to the crosslinking in the films [35], which was also confirmed by the TG and XRD results.

### 3.5. Mechanical Properties of Films

**Figure 7:**
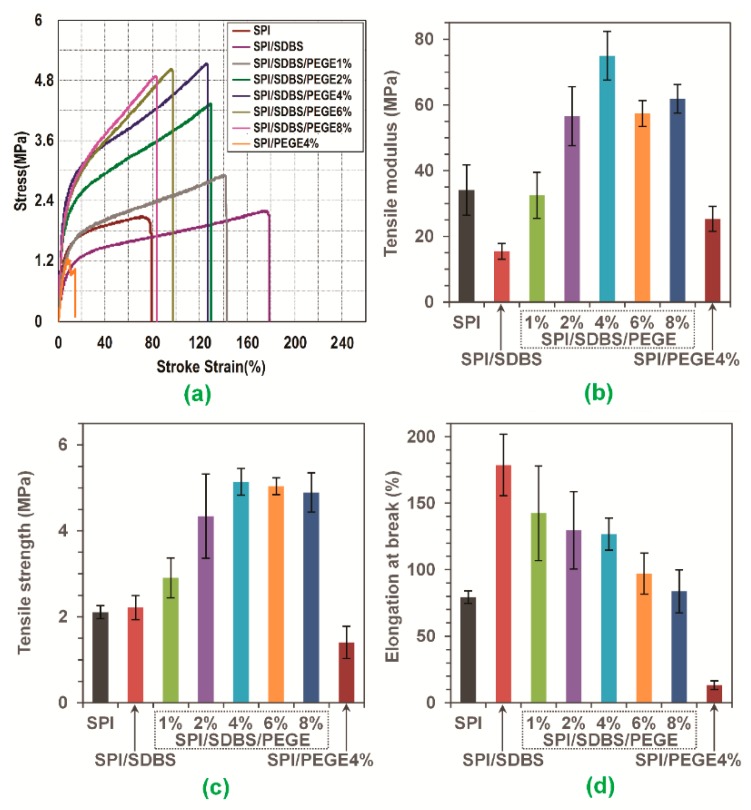
Tensile properties of the SPI-based films, including (**a**) average strain-stress curves, (**b**) tensile modulus (TE), (**c**) tensile strength (TS), and (**d**) elongation at break (EB).

The average strain-stress curves and the resulted TE, TS and EB of the films from tensile tests are shown in Figure 7. Looking at the testing results of SPI and SPI/SDBS films, it was found that TE was dramatically decreased (from 34.1 ± 7.7 to 15.4 ± 2.4 MPa), but the TS was slightly increased (from 2.1 ± 0.2 to 2.2 ± 0.3 MPa), and the EB was considerably increased (from 79.4 ± 4.7% to 178.7 ± 23.1%). It was probably due to the improvement of dispersion that made the protein chains better interaction with each other, which was evidenced by FTIR and TG. This phenomenon was similar with the SPI film with/without the addition of NaOH, therefore, SDBS could be a substitute of NaOH in producing SPI film. That will benefit to keep neutral for the SPI-based film. In Figure 7, it was found that the TE and TS of the films increased with the amount of added PEGE until reached a maximum value of 4% PEGE, and then decreased with the further addition of PEGE. EB was decreased with the addition of PEGE into SPI/SDBS. The Duncan’s multiple range test was utilized for the multiple comparison of the mechanical properties of the SPI/SDBS/PEGE films with different contents of PEGE [44], as shown in Table 4. Obviously, the SPI/SDBS/PEGE4% film presented the highest TS (5.1 ± 0.3 MPa), and TE (75.0 ± 7.5 MPa), counting the increases of 144.1% and 120.1%, respectively, in comparison with the values of the SPI film (TS = 2.1 ± 0.2 MPa, and TE = 34.1 ± 7.7 MPa). The EB of SPI/SDBS/PEGE4% film was 126.7 ± 12.1%, which was 59.7% higher than that of SPI film (EB = 79.4 ± 4.7%). The testing results of TS, TE, and EB were analyzed by ANOVA tests, confirming significant increases at level of *α* = 0.05 (*P* = 3.9 × 10^−2^, 2.4 × 10^−4^, and 1.2 × 10^−2^, respectively). These results perfectly matched with the conclusions drawn from the TG, XRD, and SEM analysis, which confirmed that the crosslinking reactions occurred between PEGE and protein chains, so that dramatically enhancing the TS and TE values of the films. The mechanical properties of SPI/PEGE4% films were obviously lower than the SPI/SDBS/PEGE4% film. It was revealed that the compatibility between protein and PEGE was substantially improved by the adding of SDBS, since the SDBS helped dispersing the hydrophobic PEGE into the hydrophilic protein matrix. This finding could also be confirmed by the macro and SEM images as shown in Figure 2 and Figure 6, respectively.

### 3.6. Water Resistance of Films

As presented in Table 5, the moisture contents and total soluble matters of the fabricated films were compared. Since glycerol was the main soluble matter in the fabricated films, the total soluble matters of the fabricated films showed no significant difference (27.5–30.1%). The moisture content of the SPI film (34.2%) was slightly reduced by 9.4% after adding SDBS (31.0% SPI/SDBS film). The moisture content was further reduced to be 28.6–23.4% after the addition of PEGE. Compared to the SPI film, the moisture content of the SPI/SDBS/PEGE4% film was significantly reduced by 22.5% at *α* = 0.05 level (ANNOVA test, *P* = 2.9 × 10^−2^). Furthermore, to evaluate the effect of different SPI/SDBS/PEGE percentages on film performance, the Duncan’s multiple range test was utilized for the multiple comparison as shown in Table 5 [44].

The 24-h water absorptions of the fabricated films are shown in Table 5, which was dramatically increased from 109.7% to 194.6% after the addition of SDBS. From our previous studies [35,45], the 24-h water absorption of the SPI film with NaOH was 209.1%, which was close to that of the SPI/SDBS film. It was further indicated that SDBS played the similar role as NaOH in the SPI film casting. The results showed that the 24-h water absorption of the SPI/SDBS/PEGE4% film was 96.9%, presenting a reduction of 50.2% compared with that of SPI/SDBS film (Table 5). It was revealed that the dramatic improvement in water-resistant property of the film was obtained due to the chemically crosslinking networks in the modified films [35,42], which could also in turn prove the existing reactions in the SPI/SDBS/PEGE films. The 24-h water absorption was gradually decreased to be 112.9%, 97.1%, 96.9%, 67.8%, and 66.4% by adding 1%, 2%, 4%, 6%, and 8% PEGE into SPI/SDBS films, respectively (Table 5). It presented an increase by the addition of more PEGE, which was different with the trend of tensile strength of the films. It was suggested that the un-reacted PEGE in the SPI/SDBS/PEGE6-8% films might service as surface protection due to their water insoluble character. The 24-h water absorption of SPI/PEGE4% film showed the lowest value (48.5%), even compared to chemical-crosslinked films, which was perfectly consistent with the hypothesis that the surface protection by PEGE serving as a barrier between water and SPI matrix, so that reducing the water absorption of the film.

## 4. Conclusions

The NaOH-free SPI-based films were successfully prepared and their properties were examined. It was revealed that SDBS could excellently play the role as NaOH on casting the SPT-based films, which could remain the resultant films neutral, so that greatly benefiting to film applications. The testing results from FTIR and TG indicated that SDBS could help homogeneously disperse proteins and PEGE and dramatically enhance their compatibility to each other. The chemical crosslinking reactions occurred between proteins and PEGE, which were confirmed by incorporating the results of the examinations of TG, XRD, SEM, mechanical properties, and water resistance of the films. The evidences of the reactions included the increased thermal stability (T_max2_ from 310.9 to 316.9 °C), decreased crystallinity (*RCI* from 31.9% to 21%), homogeneous cross-section surface, enhanced TE (from 15.4 to 75.0 MPa)/TS (from 2.2 to 5.1 MPa) and reduced EB (from 178.7% to 126.7%), and improved water resistance (24-h water absorption from 194.6% to 96.9%). This study provided the novel fabrication method of SPI-based films with effective recipe, which is helpful for film property enhancement and scale-up manufacturing.

## Figures and Tables

**Table 1:** Feedstocks for preparing SPI-based films.

Film	SPI	DI-Water	Glycerol	SDBS	PEGE	
	(g)	(mL)	(g)	(g)	(g)	(%) ^1^
SPI	4	40	2	-	-	-
SPI/SDBS	4	40	2	0.8	-	-
SPI/SDBS/PEGE1%	4	40	2	0.8	0.04	1
SPI/SDBS/PEGE2%	4	40	2	0.8	0.08	2
SPI/SDBS/PEGE4%	4	40	2	0.8	0.16	4
SPI/SDBS/PEGE6%	4	40	2	0.8	0.24	6
SPI/SDBS/PEGE8%	4	40	2	0.8	0.32	8
SPI/PEGE4%	4	40	2	-	0.16	4

^1^ Calculated by the amount of adding SPI (i.e., 4 g).

**Table 2:** TG parameters for the thermal degradation of SPI powder, SDBS, and SPI-based films.

Specimen	T_int1_^1^	T_max1_^2^	T_int2_	T_max2_	T_int3_	T_max3_
	(°C)	(°C)	(°C)	(°C)	(°C)	(°C)
SPI powder	-	-	265.3	309.8	-	-
SDBS	-	-	-	-	448.2	464.0
SPI	134.8	152.2	174.5	303.2	-	-
SPI/SDBS	135.8	163.3	188.0	310.9	459.4	480.5
SPI/SDBS/PEGE4%	135.2	162.2	185.7	316.9	465.6	483.5
SPI/PEGE4%	133.7	156.4	173.8	311.0	-	-

^1^ T_int_ = temperature of initial decomposition; ^2^ T_max_ = peak temperature.

**Table 3:** Crystallinity of the SPI powder, SDBS and SPI-based films.

Specimen	*RCI* ^1^
	(%)
SPI powder	30.1 ± 0.7 ^2^
SDBS	28.7 ± 0.4
SPI	29.5 ± 0.9
SPI/SDBS	31.9 ± 0.7
SPI/SDBS/PEGE4%	21.0 ± 0.4
SPI/PEGE4%	29.4 ± 0.7

^1^*RCI* = relative crystallinity index; ^2^ Mean ± standard deviation.

**Table 4:** A comparison of tensile modulus, strength and elongation at break of the SPI/SDBS/PEGE films by adding various amounts of PEGE.

PEGE Content	Tensile Modulus	Tensile Strength	Elongation at Break
(%)	(MPa)	(MPa)	(%)
0	15.4 ± 2.4 ^1^ (A) ^2^	2.2 ± 0.3 (A)	178.7 ± 23.1 (A)
1	32.5 ± 7.0 (B)	2.9 ± 0.5 (A)	142.5 ± 35.5 (AB)
2	56.6 ± 9.0 (C)	4.3 ± 1.0 (B)	129.7 ± 29.1 (BC)
4	75.0 ± 7.5 (D)	5.1 ± 0.3 (B)	126.7 ± 12.1 (BC)
6	57.4 ± 3.9 (C)	5.0 ± 0.2 (B)	97.2 ± 15.4 (CD)
8	61.9 ± 4.4 (C)	4.9 ± 0.5 (B)	83.7 ± 16.2 (D)

^1^ Mean ± standard deviation; ^2^ Groups with the same letters in each column indicate that there is no statistical difference (*p* < 0.05) between the samples according to the Duncan’s multiple range test. The detail results are presented in Supplemental Information Tables S1–S3.

**Table 5:** Moisture content after conditioning, water absorption and total soluble matter after 24 h submersion of the SPI-based films.

Film	Moisture Content	24-h Water Absorption	Total Soluble Matter
	(%)	(%)	(%)
SPI	34.2 ± 2.6 ^1^	109.7 ± 11.0 (B) ^2^	27.5 ± 0.5 (AB)
SPI/SDBS	31.0 ± 1.3	194.6 ± 11.1 (A)	27.6 ± 0.6 (AB)
SPI/SDBS/PEGE1%	28.6 ± 1.7	112.9 ± 10.0 (B)	30.1 ± 1.0 (A)
SPI/SDBS/PEGE2%	26.7 ± 2.3	97.1 ± 10.2 (BC)	28.3 ± 1.5 (AB)
SPI/SDBS/PEGE4%	26.5 ± 2.0	96.9 ± 6.8 (BC)	26.4 ± 1.6 (B)
SPI/SDBS/PEGE6%	26.6 ± 2.0	67.8 ± 3.8 (BC)	29.1 ± 1.2 (AB)
SPI/SDBS/PEGE8%	23.4 ± 2.0	66.4 ± 3.8 (BC)	28.6 ± 2.8 (AB)
SPI/PEGE4%	29.5 ± 1.9	48.5 ± 3.1 (C)	27.8 ± 1.4 (AB)

^1^ Mean ± standard deviation; ^2^ Groups with the same letters in each column indicate that there is no statistical difference (*p* < 0.05) between the samples according to the Duncan’s multiple range test. The detail results are presented in Supplemental Information Tables S4 and S5.

## Supplementary Materials

The following are available online at http://www.mdpi.com/2073-4360/10/12/1300/s1, Table S1: Statistical analysis of tensile strength according to the Duncan’s multiple range test, Table S2: Statistical analysis of tensile modulus according to the Duncan’s multiple range test, Table S3: Statistical analysis of tensile elongation at break according to the Duncan’s multiple range test, Table S4: Statistical analysis of 24-h water absorption according to the Duncan’s multiple range test, Table S5: Statistical analysis of total soluble matter according to the Duncan’s multiple range test.
