# Supplementary material for: Sodium Hydroxide-Free Soy Protein Isolate-Based Films Crosslinked by Pentaerythritol Glycidyl Ether
Source: Polymers (Basel). 2018 Nov 23;10(12):1300. doi: 10.3390/polym10121300 (PMC6401677; doi:10.3390/polym10121300)
Supplement: Supplementary file 1 [file polymers-10-01300-s001.pdf]

# Sodium Hydroxide-Free Soy Protein Isolate-Based Films Crosslinked by Pentaerythritol Glycidyl Ether

**Yingji Wu [1], Liping Cai [1,2], Chen Wang [3], Changtong Mei [1,*] and Sheldon Q. Shi [2,*]**

[1] College of Materials Science and Engineering, Nanjing Forestry University, Nanjing, Jiangsu 210037, China; wuyingji@njfu.edu.cn

[2] Department of Mechanical and Energy Engineering, University of North Texas, Denton, TX 76203, USA; liping.cai@unt.edu

[3] Research Institute of Wood Industry, Chinese Academy of Forestry, Beijing 100091, China; wangchen0903@163.com

* Correspondence: mei@njfu.edu.cn (C.M.); sheldon.shi@unt.edu (S.Q.S.); Tel.: +1-940-369-5930 (S.Q.S.)

## Supplemental Information

**Table S1.** Statistical analysis of tensile strength according to the Duncan's multiple range test.

| Category | LS means | Standard error | Lower bound (95%) | Upper bound (95%) | Groups |
|---|---|---|---|---|---|
| SPI/SDBS | 15.356 | 4.086 | 6.436 | 23.676 | A |
| SPI/SDBS/PEGE1% | 32.456 | 3.538 | 24.991 | 39.922 | B |
| SPI/SDBS/PEGE2% | 56.567 | 3.538 | 49.102 | 64.032 | C |
| SPI/SDBS/PEGE6% | 57.381 | 3.538 | 49.916 | 64.846 | C |
| SPI/SDBS/PEGE8% | 61.864 | 3.538 | 54.399 | 69.329 | C |
| SPI/SDBS/PEGE4% | 74.971 | 3.538 | 67.505 | 82.436 | D |

**Table S2.** Statistical analysis of tensile modulus according to the Duncan's multiple range test.

| Category | LS means | Standard error | Lower bound (95%) | Upper bound (95%) | Group s |
|---|---|---|---|---|---|
| SPI/SDBS | 2.168 | 0.354 | 1.421 | 2.915 | A |
| SPI/SDBS/PEGE1% | 2.953 | 0.307 | 2.306 | 3.599 | A |
| SPI/SDBS/PEGE2% | 4.342 | 0.307 | 3.696 | 4.989 | B |
| SPI/SDBS/PEGE8% | 4.895 | 0.307 | 4.248 | 5.541 | B |
| SPI/SDBS/PEGE6% | 5.039 | 0.307 | 4.392 | 5.686 | B |
| SPI/SDBS/PEGE4% | 5.140 | 0.307 | 4.493 | 5.787 | B |

**Table S3.** Statistical analysis of tensile elongation at break according to the Duncan's multiple range test.

| Category | LS means | Standard error | Lower bound (95%) | Upper bound (95%) | Groups |
|---|---|---|---|---|---|
| SPI/SDBS | 178.669 | 15.819 | 151.344 | 207.972 | A |
| SPI/SDBS/PEGE1% | 142.465 | 13.700 | 113.562 | 171.368 | A B |
| SPI/SDBS/PEGE2% | 129.703 | 13.700 | 100.800 | 158.606 | B C |
| SPI/SDBS/PEGE4% | 126.749 | 13.700 | 97.846 | 155.653 | B C |
| SPI/SDBS/PEGE6% | 97.173 | 13.700 | 68.270 | 126.077 | C D |
| SPI/SDBS/PEGE8% | 83.716 | 13.700 | 54.812 | 112.619 | D |

**Table S4.** Statistical analysis of 24-h water absorption according to the Duncan's multiple range test.

| Category | LS means | Standard error | Lower bound (95%) | Upper bound (95%) | Groups | |
|---|---|---|---|---|---|---|
| SPI/SDBS | 1.946 | 0.122 | 1.686 | 2.206 | A | |
| SPI | 1.097 | 0.149 | 0.848 | 1.486 | B | |
| SPI/SDBS/PEGE1% | 1.129 | 0.122 | 0.869 | 1.389 | B | |
| SPI/SDBS/PEGE2% | 0.971 | 0.122 | 0.711 | 1.232 | B | C |
| SPI/SDBS/PEGE4% | 0.969 | 0.122 | 0.709 | 1.229 | B | C |
| SPI/SDBS/PEGE6% | 0.678 | 0.122 | 0.418 | 0.938 | B | C |
| SPI/SDBS/PEGE8% | 0.664 | 0.122 | 0.403 | 0.924 | B | C |
| SPI/PEGE4% | 0.485 | 0.122 | 0.225 | 0.745 | | C |

**Table S5.** Statistical analysis of total soluble matter according to the Duncan's multiple range test.

| **Category** | **LS means** | **Standard error** | **Lower bound (95%)** | **Upper bound (95%)** | **Groups** | |
|---|---|---|---|---|---|---|
| SPI/SDBS/PEGE1% | 0.301 | 0.011 | 0.277 | 0.324 | A | |
| SPI/SDBS/PEGE6% | 0.291 | 0.011 | 0.267 | 0.314 | A | B |
| SPI/SDBS/PEGE8% | 0.286 | 0.011 | 0.262 | 0.309 | A | B |
| SPI/SDBS/PEGE2% | 0.283 | 0.011 | 0.259 | 0.306 | A | B |
| SPI/PEGE4% | 0.278 | 0.011 | 0.255 | 0.301 | A | B |
| SPI/SDBS | 0.276 | 0.011 | 0.253 | 0.299 | A | B |
| SPI | 0.275 | 0.013 | 0.246 | 0.303 | A | B |
| SPI/SDBS/PEGE4% | 0.264 | 0.011 | 0.240 | 0.287 | | B |
